# Pulmonary alveolar proteinosis diagnosed during leuprorelin acetate therapy for ovarian endometrioma: a case report

**DOI:** 10.1186/s12890-026-04507-5

**Published:** 2026-07-17

**Authors:** Xin Gao, Lei Wang, Zhiwei Li, Jing Zhou, Fuqiang Ren

**Affiliations:** Department of Cardiothoracic Surgery, Chengdu Sixth People’s Hospital, No. 369 Quanshui Road, Jinniu District, Chengdu, 610000 China

**Keywords:** Pulmonary alveolar proteinosis, Gonadotropin-releasing hormone agonist, Leuprorelin acetate, Ovarian endometrioma, Drug-induced lung disease

## Abstract

**Background:**

Pulmonary alveolar proteinosis (PAP) is a rare pulmonary disorder characterized by impaired surfactant clearance due to alveolar macrophage dysfunction. Leuprorelin acetate, a gonadotropin-releasing hormone agonist, is widely used in the treatment of ovarian endometriosis; however, pulmonary findings associated with this therapy have not been well characterized. We report a case of PAP diagnosed during leuprorelin acetate therapy for ovarian endometrioma and describe its temporal relationship with treatment.

**Case presentation:**

A 29-year-old woman underwent laparoscopic surgery for an ovarian endometrioma in August 2025 and initiated leuprorelin acetate therapy three days postoperatively. A routine chest computed tomography (CT) performed one month later revealed scattered bilateral ground-glass opacities (GGOs), which persisted on repeat CT in November 2025. Although the patient remained asymptomatic, video-assisted thoracoscopic surgery (VATS) lung biopsy was performed to obtain a histopathological diagnosis and confirmed PAP. Serum anti-granulocyte-macrophage colony-stimulating factor (GM-CSF) antibody testing was negative (3.2 µg/mL; reference range, ≤ 5 µg/mL), not supporting autoimmune PAP. During treatment, the patient developed transient Common Terminology Criteria for Adverse Events (CTCAE) grade 2 leukopenia (white blood cell nadir, 2.94 × 10⁹/L), which resolved spontaneously after completion of leuprorelin acetate therapy without intervention. Hematological evaluation did not suggest an underlying clonal disorder, including myelodysplastic syndrome. Because baseline chest imaging was unavailable, pre-existing subclinical PAP could not be excluded. Follow-up CT performed three months after completion of therapy showed regression of GGOs.

**Conclusion:**

This case shows a temporal relationship between leuprorelin acetate therapy and non-autoimmune PAP. However, causality cannot be inferred in the absence of baseline imaging. Only a temporal association can be observed. This observation is hypothesis-generating and highlights a potential rare pulmonary finding observed in the context of gonadotropin-releasing hormone agonist therapy.

## Introduction

Pulmonary alveolar proteinosis (PAP) is a rare diffuse interstitial lung disease characterized by impaired clearance of pulmonary surfactant due to dysfunction of alveolar macrophages or disruption of granulocyte–macrophage colony-stimulating factor (GM-CSF) signaling pathways [1, 2]. Based on its underlying pathogenesis, PAP is classified into three types: autoimmune, secondary, and congenital [2]. Autoimmune PAP is caused by elevated anti–GM-CSF antibodies that neutralize GM-CSF signaling, whereas secondary PAP results from impaired alveolar macrophage function associated with hematological malignancies, immunosuppressive therapy, chronic infections, organ transplantation, or inhalational exposure. Congenital PAP is caused by genetic mutations affecting surfactant production or metabolism [2, 3].

The clinical manifestations of PAP typically include progressive dyspnea, dry cough, and fatigue. In some patients, secondary pulmonary infections may occur, and severe disease may progress to respiratory failure. For asymptomatic or mild disease, close observation with regular follow-up is recommended. In moderate-to-severe cases, treatment aims to improve oxygenation, relieve symptoms, and improve quality of life. Whole-lung lavage remains the standard treatment for severe disease. In selected patients with autoimmune PAP, GM-CSF replacement therapy via inhaled or subcutaneous routes may also be considered [2, 4, 5]. Leuprorelin acetate is widely used as postoperative adjuvant therapy for endometriosis because of its proven efficacy in reducing disease recurrence and improving reproductive outcomes [6].

Herein, we report a case of a patient with ovarian endometrioma who developed multiple ground-glass opacities (GGOs) on chest computed tomography (CT) during treatment with leuprorelin acetate and was ultimately diagnosed with PAP.

## Case presentation

### Patient information

A 29-year-old woman working as a nurse presented to our department in November 2025 with persistent pulmonary opacities identified on imaging. She had no history of occupational or environmental exposure to silica, toxic dust, or aerosolized medications. Her medical history was notable for two previous surgeries for ovarian endometrioma. In August 2025, three days after her second surgery, she initiated treatment with leuprorelin acetate (3.75 mg, subcutaneously every 28 days). She was a lifelong non-smoker and had a history of allergy to penicillin and cephalosporins.

### Clinical findings

The patient remained completely asymptomatic, with no cough, sputum production, or dyspnea. In September 2025, one month after initiation of leuprorelin acetate, a routine chest CT scan incidentally revealed scattered GGOs involving multiple lobes (Fig. 1a–b). A repeat CT scan in November 2025 demonstrated persistence of these lesions, predominantly distributed along the peribronchovascular regions with focal interstitial thickening. Typical “crazy-paving” or geographic patterns commonly described in pulmonary alveolar proteinosis were not observed. Physical examination on admission was unremarkable, with clear breath sounds bilaterally and a peripheral oxygen saturation of 99% on room air.

**Fig. 1:**
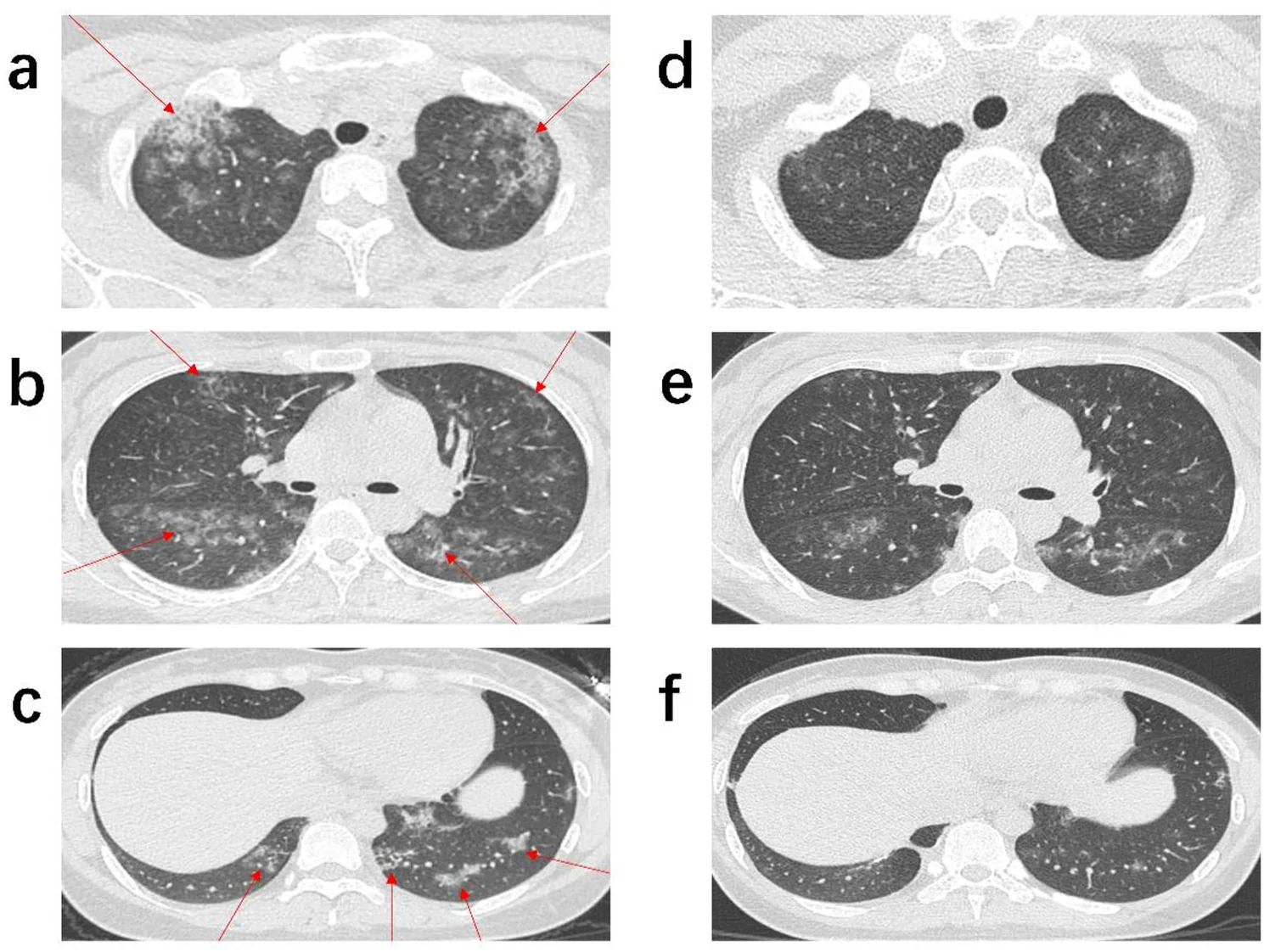
Scattered ground-glass opacities (GGOs) in both lungs **a**–**c**. Three-month follow-up chest CT after discontinuation of leuprorelin acetate demonstrated marked interval regression of the bilateral GGOs **d**–**f**

### Diagnostic assessment

Laboratory tests revealed a white blood cell (WBC) count of 2.94 × 10⁹/L and an absolute lymphocyte count of 0.83 × 10⁹/L, consistent with Common Terminology Criteria for Adverse Events (CTCAE) grade 2 leukopenia. Serum anti–GM-CSF antibody testing was performed using an enzyme-linked immunosorbent assay (ELISA), with a result of 3.2 µg/mL (reference range, ≤ 5 µg/mL), making autoimmune pulmonary alveolar proteinosis unlikely based on available serological evidence. Liver and renal function tests, coagulation profiles, and tumor markers were all within normal limits. The interferon-gamma release assay for tuberculosis (TB-IGRA) was negative. The laboratory results are summarized in Table 1.

**Table 1:** Summary of the patient’s laboratory data

	On admission	Reference range
WBC (*10^9^/L)	2.94	3.5–9.5
RBC (*10^12^/L)	4.82	3.8–5.1
HGB (g/L)	150	115–150
PLT (*10^9^/L)	153	125–350
CRP (mg/L)	0.2	0–10
D-dimer (mg/L)	0.15	0-0.55
TP (g/L)	78.6	65–85
Alb (g/L)	47.5	40–55
AST (U/L)	21	13–35
ALT (U/L)	12	7–40
ALP (U/L)	78	35–100
T-Bil (µmol/L)	2.3	0–23
Urea (mmol/L)	5.7	2.6–7.5
Cre (µmol/L)	51.8	41–73
Na (mmol/L)	138.1	137–147
K (mmol/L)	4.86	3.5–5.5
Cl (mmol/L)	101.3	99–110
CEA (ng/mL)	1.0	0–5
CYFRA (ng/mL)	1.84	0-3.3
ESR (mm/h)	11.0	0–20
TB-IGRA (pg/ml)	0.30	0–14
Anti-CCP (U/mL)	<8	<17
AKA	(-)	(-)
ASO (IU/mL)	114	≤ 200
C3 (g/L)	0.92	0.7–1.4
C4 (g/L)	0.22	0.1–0.4
IgE (IU/mL)	183	<358
IgA (g/L)	2.51	1.0-4.2
IgG (g/L)	13.87	8.6–17.4
IgM (g/L)	1.36	0.5–2.8
RF (IU/mL)	<10.0	<14.0
BPI	<1.0	<1.0
CG	<1.0	<1.0
HLE	<1.0	<1.0
LF	<1.0	<1.0
MPO	<1.0	<1.0
PR3	<1.0	<1.0
ANA	(-)	(-)
Anti-dsDNA (IU/mL)	7.8	<30.0
Anti-Sm (AI)	4.9	<10.0
Anti-SSA (AI)	2.9	<10.0
Anti-Ro52 (AI)	3.4	<10.0
Anti-SSB (AI)	4.2	<10.0
Anti-Scl70 (AI)	2.9	<10.0
Anti-P0 (AI)	6.1	<10.0
anti-PM-Scl (AI)	2.7	<10.0
Anti-CENP-B (AI)	2.0	<10.0
Anti-Jo-1 (AI)	3.8	<10.0
Anti-U1-RNP (AI)	4.5	<10.0
GM-CSF(µg/ml)	3.2	≤ 5

*WBC* White blood cell count, *RBC* Red blood cell count, *HGB* Hemoglobin, *PLT* Platelet count, *CRP* C-reactive protein, *TP* Total protein, *Alb* Albumin, *AST* Aspartate aminotransferase, *ALT* Alanine aminotransferase, *ALP* Alkaline phosphatase, *T-Bil* Total bilirubin, *Urea* Urea, *Cre* Creatinine, *Na* Sodium, *K* Potassium, *Cl* Chloride, *CEA* Carcinoembryonic antigen, *CYFRA* Cytokeratin 19 fragment (CYFRA 21 − 1), *ESR* Erythrocyte sedimentation rate, *TB-IGRA* Tuberculosis interferon-gamma release assay, *Anti-CCP* Anti-cyclic citrullinated peptide antibody, *AKA* Anti-keratin antibody, *ASO* Antistreptolysin O, *C3* Complement component 3, *C4* Complement component 4, *IgE* Immunoglobulin E, *IgA* Immunoglobulin A, *IgG* Immunoglobulin G, *IgM* Immunoglobulin M, *RF* Rheumatoid factor, *BPI* Bactericidal/permeability-increasing protein, *CG* Cathepsin G, *HLE* Human leukocyte elastase, *LF* Lactoferrin, *MPO* Myeloperoxidase, *PR3* Proteinase 3, *ANA* Antinuclear antibody, *Anti-dsDNA* Anti-double stranded DNA antibody, *Anti-Sm* Anti-Smith antibody, *Anti-SSA* Anti-Sjögren’s-syndrome-related antigen A antibody (anti-Ro), Anti*-Ro52* Anti-Ro52 antibody, *Anti-SSB* Anti-Sjögren’s-syndrome-related antigen B antibody (anti-La), *Anti-Scl70* Anti-scleroderma 70 antibody (anti-topoisomerase I), *Anti-P0* Anti-ribosomal P0 antibody, *Anti-PM-Scl* Anti-polymyositis/scleroderma antibody, *Anti-CENP-B* Anti-centromere protein B antibody, *Anti-Jo-1* Anti-histidyl-tRNA synthetase antibody, *Anti-U1-RNP* Anti-U1 small nuclear ribonucleoprotein antibody, *GM-CSF* Granulocyte-macrophage colony-stimulating factor

Given the patient’s stable clinical condition and normal oxygen saturation, arterial blood gas analysis and pulmonary function testing were not performed. Although bronchoalveolar lavage (BAL) is often used in the diagnostic evaluation of pulmonary alveolar proteinosis, the clinical team proceeded directly to video-assisted thoracoscopic surgery (VATS) wedge biopsy of the right lower lobe (Fig. 2a-b), aiming to obtain a definitive histopathological diagnosis and to exclude other interstitial lung diseases or occult malignancy in the context of non-specific imaging findings.

**Fig. 2:**
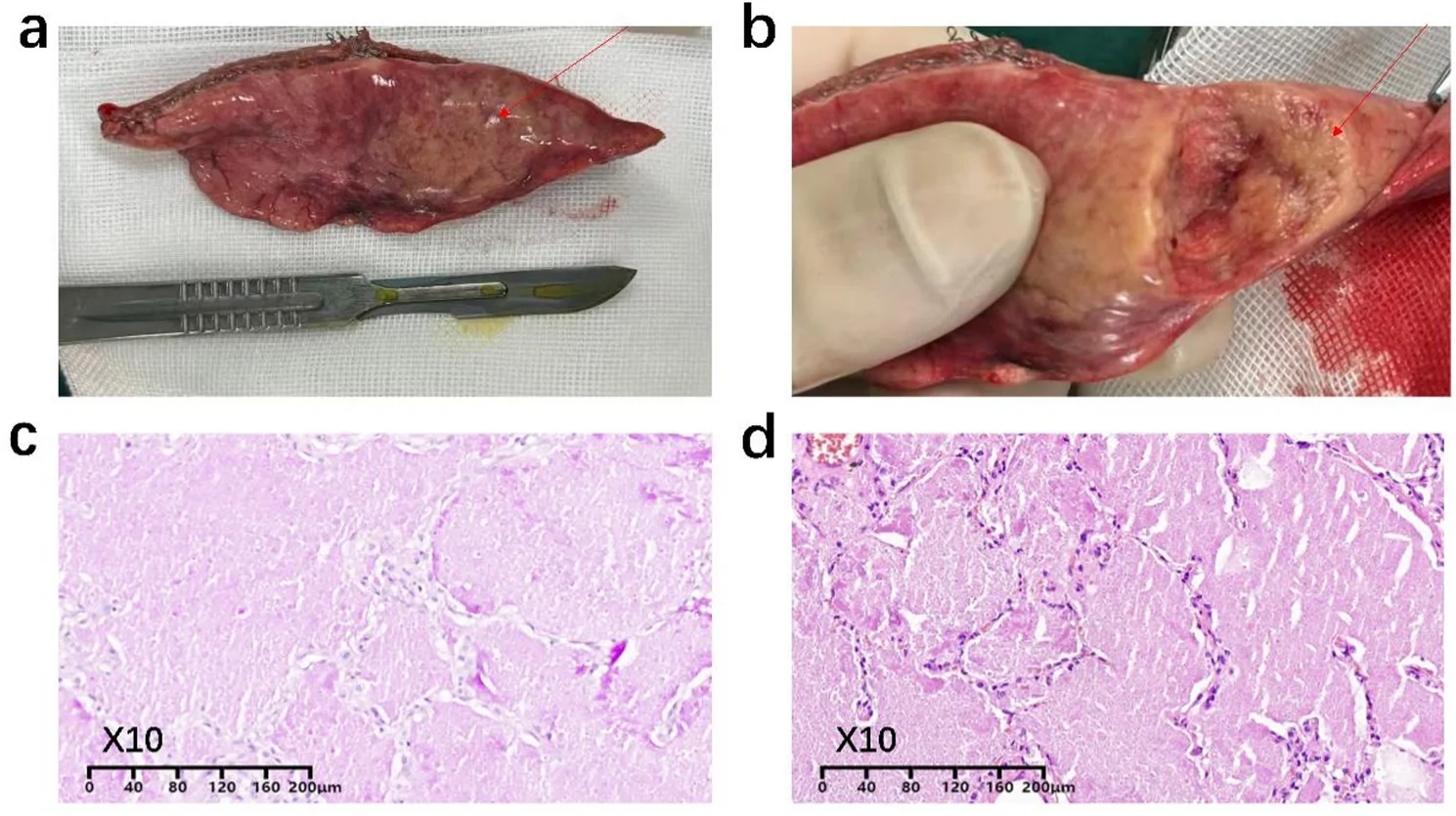
The lung parenchyma at the site of alveolar protein deposition appeared gray-white **a**–**b**. Histopathological examination revealed eosinophilic granular material filling the alveolar spaces on hematoxylin and eosin (H&E) staining **c**. The same material showed positive periodic acid–Schiff (PAS) staining **d**

Histopathological examination demonstrated eosinophilic granular material filling the alveolar spaces on hematoxylin and eosin (H&E) staining (Fig. 2c). The same material showed strong periodic acid–Schiff (PAS) positivity (Fig. 2d), consistent with pulmonary alveolar proteinosis. The patient was diagnosed with mild non-autoimmune pulmonary alveolar proteinosis, and no specific therapeutic intervention was initiated due to the absence of symptoms or hypoxemia.

### Follow-up and outcomes

The patient was managed conservatively with observation. Leuprorelin acetate therapy was completed in March 2026, and spontaneous resumption of menstruation occurred in May 2026. A follow-up chest CT performed in May 2026 demonstrated a significant reduction in the previously observed ground-glass opacities (Fig. 1d-f).

During six months of follow-up, serial blood tests showed normalization and sustained stability of the white blood cell count without intervention. This spontaneous recovery, in the absence of hematologic treatment, suggested that the leukopenia was transient and likely related to leuprorelin acetate exposure rather than an underlying clonal hematologic disorder such as myelodysplastic syndrome.

## Discussion and conclusion

This case describes a patient with ovarian endometrioma who was diagnosed with PAP shortly after initiation of postoperative leuprorelin acetate therapy. Although the absence of baseline chest imaging precludes determination of the exact onset of PAP, the temporal relationship between treatment initiation and the subsequent radiological findings, together with spontaneous radiological improvement after completion of leuprorelin acetate therapy, represents a clinical observation.

Accurate classification of PAP is essential because the underlying etiology has important implications for clinical management [2]. Approximately 90% of PAP cases are autoimmune and are associated with circulating anti–GM-CSF antibodies that impair alveolar macrophage function [2]. In the present case, serum anti–GM-CSF antibody testing by ELISA showed a level of 3.2 µg/mL (reference range, ≤ 5 µg/mL), making autoimmune PAP unlikely based on the available serological evidence. Secondary PAP is most commonly associated with hematological disorders, immunodeficiency, chronic infections, or inhalational exposures [2, 3]. Although the patient developed transient CTCAE grade 2 leukopenia during treatment, serial blood counts normalized spontaneously after completion of therapy and remained stable throughout follow-up, making an underlying clonal hematological disorder unlikely based on the available data. In addition, there was no history of occupational or environmental exposure known to be associated with secondary PAP.

To the best of our knowledge, only limited evidence has suggested a potential association between leuprorelin acetate and PAP. Nevertheless, gonadotropin-releasing hormone (GnRH) agonists are known to influence immune function through interactions between the endocrine and immune systems [7, 8].Experimental and clinical studies have shown that GnRH agonists can alter immune cell populations, including T lymphocytes and monocyte/macrophage lineages [9–12], although the direction and clinical significance of these effects appear to vary according to the physiological and disease context [13]. Leuprorelin acetate is widely used as an adjuvant postoperative therapy for endometriosis because of its well-established efficacy in reducing disease recurrence and improving reproductive outcomes, making recognition of uncommon adverse events clinically important [6]. Whether such immunological changes contribute to impaired surfactant clearance has not been clearly elucidated.

Another possible explanation involves the profound hypoestrogenic state induced by prolonged GnRH agonist therapy. Beyond its reproductive functions, accumulating evidence indicates that estrogen signaling is an important regulator of myeloid cell biology and macrophage immune responses [14, 15]. Experimental evidence suggests that estrogen contributes to the regulation of macrophage differentiation, activation, and phagocytic function [15, 16]. Estrogen deprivation has been associated with reduced macrophage phagocytic activity and altered inflammatory responses in experimental models [16–18], while clinical studies of ovarian suppression have also demonstrated systemic immune alterations [19]. Although these observations do not establish a direct relationship with PAP, they provide a biologically plausible framework for future investigation.

Based on the available evidence, we consider this case to represent a hypothesis-generating temporal association rather than evidence of causality. One possible explanation is that, in individuals with unrecognized susceptibility affecting alveolar macrophage function or surfactant homeostasis, the combined effects of profound hypoestrogenism and transient immune modulation during leuprorelin acetate therapy may have been temporally associated with the development or unmasking of PAP. However, this hypothesis remains speculative and requires further confirmation through additional clinical observations and mechanistic studies.

## Conclusion

This report describes a patient who developed non-autoimmune pulmonary alveolar proteinosis during postoperative leuprorelin acetate therapy for ovarian endometrioma. Because baseline chest imaging was unavailable, a causal relationship between leuprorelin acetate and PAP cannot be established, and only a temporal association can be suggested based on the current evidence. Nevertheless, the spontaneous radiological improvement observed after completion of therapy, in the absence of clear alternative explanations, may represent a previously unreported and incidental finding. This case may increase clinical awareness of a potential pulmonary adverse event associated with GnRH agonists and provides a basis for future clinical and mechanistic studies investigating the effects of hypoestrogenism and immune modulation on alveolar macrophage function.

## Data Availability

The data supporting the findings of this study are available from the corresponding author on reasonable request.

## Abbreviations

AKA: Anti-keratin antibody
Alb: Albumin
ALP: Alkaline phosphatase
ALT: Alanine aminotransferase
ANA: Antinuclear antibody
Anti-CCP: Anti-cyclic citrullinated peptide antibody
Anti-CENP-B: Anti-centromere protein B antibody
Anti-dsDNA: Anti–double-stranded DNA antibody
Anti-Jo-1: Anti–histidyl-tRNA synthetase antibody
Anti-P0: Anti-ribosomal P0 antibody
Anti-PM-Scl: Anti–polymyositis/scleroderma antibody
Anti-Ro52: Anti-Ro52 antibody
Anti-Scl70: Anti–scleroderma 70 antibody (anti–topoisomerase I)
Anti-Sm: Anti-Smith antibody
Anti-SSA: Anti–Sjögren’s-syndrome-related antigen A antibody (anti-Ro)
Anti-SSB: Anti–Sjögren’s-syndrome-related antigen B antibody (anti-La)
Anti-U1-RNP: Anti–U1 small nuclear ribonucleoprotein antibody
ASO: Antistreptolysin O
AST: Aspartate aminotransferase
BAL: Bronchoalveolar lavage
BPI: Bactericidal/permeability-increasing protein
C3: Complement component 3
C4: Complement component 4
CEA: Carcinoembryonic antigen
CG: Cathepsin G
Cl: Chloride
Cre: Creatinine
CRP: C-reactive protein
CT: Computed tomography
CTCAE: Common Terminology Criteria for Adverse Events
CYFRA: Cytokeratin 19 fragment (CYFRA 21-1)
ELISA: Enzyme-linked immunosorbent assay
ESR: Erythrocyte sedimentation rate
GGOs: Ground-glass opacities
GM-CSF: Granulocyte-macrophage colony-stimulating factor
GnRH agonist: Gonadotropin-releasing hormone agonist
HBG: Hemoglobin
H&E: Hematoxylin and eosin
IgA: Immunoglobulin A
IgG: Immunoglobulin G
IgM: Immunoglobulin M
IgE: Immunoglobulin E
K: Potassium
LF: Lactoferrin
MPO: Myeloperoxidase
Na: Sodium
PAP: Pulmonary alveolar proteinosis
PAS: Periodic acid-Schiff
PLT: Platelet count
PR3: Proteinase 3
RBC: Red blood cell count
RF: Rheumatoid factor
T-Bil: Total bilirubin
TB-IGRA: Tuberculosis interferon-gamma release assay
TP: Total protein
Urea: Urea
VATS: Video-assisted thoracoscopic surgery
WBC: White blood cell count
